# Supplementary figures and images for: Visual Salience Controls the Speed of Evidence Accumulation in Value-Based Decisions by Rats
Source: eNeuro. 2026 Jun 19;13(6):ENEURO.0397-25.2026. doi: 10.1523/ENEURO.0397-25.2026 (PMC13421839; doi:10.1523/ENEURO.0397-25.2026)

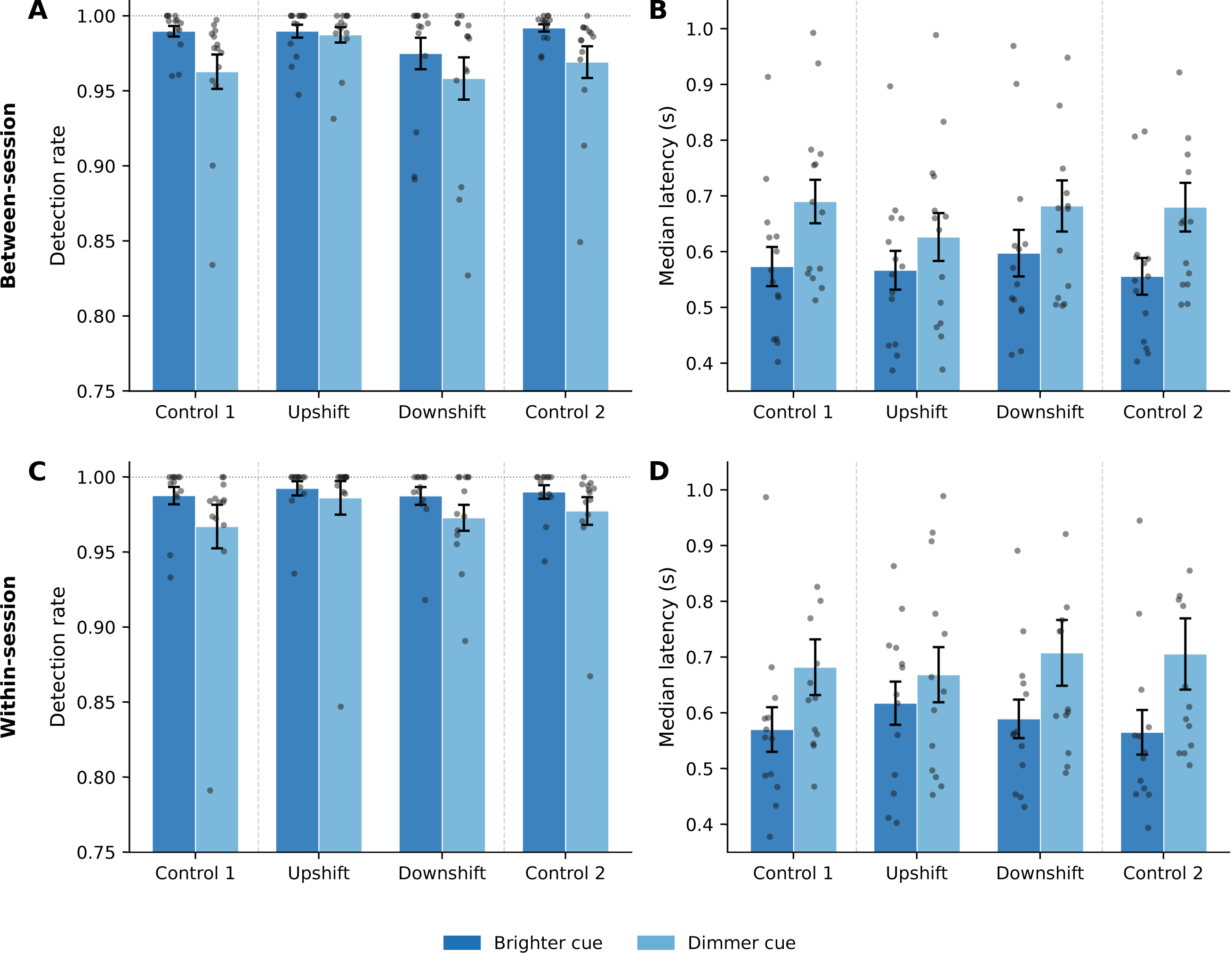

Supplement: Figure 2-1 — Single-offer trial performance was unaffected by luminance shifts. Detection rates and median latencies for single-offer trials are shown for control sessions (Control 1 and Control 2), upshift sessions (16 vs 4 LEDs), and downshift sessions (4 vs 1 LEDs). Brighter cue (dark blue) and dimmer cue (light blue) are shown as separate bars for each session type. A. Between-session detection rates. Detection remained near the ceiling across all session types for both cues, with no significant effect of session on detection in control sessions (p = 0.061) and no significant difference between brighter and dimmer cues within shift sessions (both p > 0.40). B. Between-session median latencies. No significant session effect was observed in control sessions (p = 0.35). Animals responded faster to the brighter cue in upshift sessions (p = 0.010), consistent with the luminance-driven latency advantage seen in dual-offer trials. C. Within-session detection rates (post-shift period). There was no significant effect in control sessions (p = 0.088) and no significant brighter-versus-dimmer difference in upshift sessions (p = 0.39). A significant brighter-versus-dimmer difference was present in downshift sessions (p = 0.006), indicating that animals could detect the 4-LED versus 1-LED distinction. D. Within-session median latencies (post-shift period). No significant session effect was found in the control sessions (p = 0.44). Significant brighter-versus-dimmer latency differences were present in downshift sessions (p = 0.002). Individual animal values are shown as dots. Error bars indicate SEM. Download Figure 2-1, TIF file. [file eneuro-13-ENEURO.0397-25.2026-s002.tif]

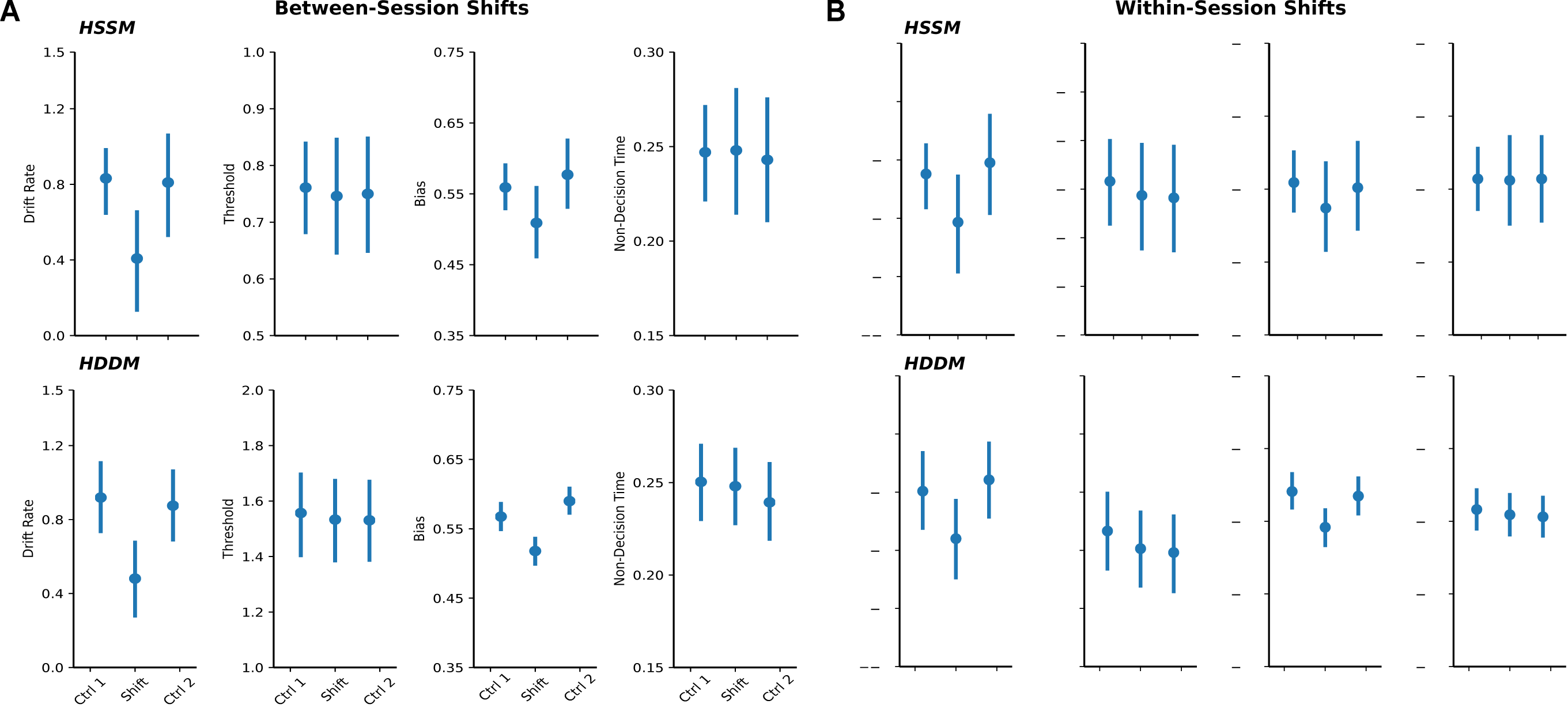

Supplement: Figure 3-1 — Comparison of HSSM and HDDM drift diffusion model parameter estimates. Results are shown for between-session shifts (A) and within-session shifts (B), collapsed across upshift and downshift directions. Each panel shows posterior means and highest density intervals (HDIs) for control sessions (Ctrl 1, Ctrl 2) and shift sessions across four DDM parameters: drift rate, decision threshold, starting point bias, and non-decision time. Top rows: HSSM (v0.3.0) estimates with 94% HDIs. Bottom rows: HDDM (v0.9.6) estimates with 95% credible intervals. Both packages show consistent reductions in drift rate and starting point bias during shift sessions, with threshold and non-decision time unaffected. Note that threshold estimates differ in absolute scale between packages due to differences in parameterization, but the pattern of effects is consistent. Download Figure 3-1, TIF file. [file eneuro-13-ENEURO.0397-25.2026-s003.tif]
